# Metabolomic signatures for liver tissue and cecum contents in high-fat diet-induced obese mice based on UHPLC-Q-TOF/MS

**DOI:** 10.1186/s12986-021-00595-8

**Published:** 2021-06-30

**Authors:** Hongying Cai, Zhiguo Wen, Kun Meng, Peilong Yang

**Affiliations:** 1 Institute of Feed Research, Chinese Academy of Agricultural Sciences, No. 12 Zhongguancun South Street, Beijing, 100081 People’s Republic of China; 2 National Engineering Research Center of Biological Feed, Beijing, 100081 People’s Republic of China

**Keywords:** Obesity, Metabolomics, UHPLC-Q-TOF/MS, Metabolic profiles, Biomarker, High-fat diet

## Abstract

**Background:**

The incidence of obesity is increasing worldwide, and it is a risk factor for diabetes, dyslipidemia, and nonalcoholic fatty liver disease. Our previous study had demonstrated that high-fat diet induced increased weight gain, fat weight, serum cholesterol, triglyceride, and ATL levels in liver, and influenced the diversity and composition of cecal microbiota in mice. Hence, this study aimed to investigate the roles of the gut microbially derived metabolites and liver metabolites between the obese and lean mice, focusing on their association with the progression of obesity induced by high-fat diet (HFD).

**Methods:**

An obesity model in mice was established with HFD for 16 weeks. Cecal contents and liver tissues metabolomics based on ultraperformance liquid chromatography-quadrupole-time-of-flight mass spectrometry and orthogonal partial least squares discriminant analyses (OPLS-DA) was performed to identify the alterations in metabolites associated with obese mice.

**Results:**

Obese and lean groups were clearly discriminated from each other on OPLS-DA score plot and major metabolites contributing to the discrimination were mainly involved in glycerophospholipid metabolism, primary bile acid biosynthesis, and biosynthesis of unsaturated fatty acids pathways. HFD-induced alterations of 19 metabolites in liver and 43 metabolites in cecum contents were identified as potential biomarkers related to obesity. Specifically, chenodeoxycholic acid, taurochenodeoxycholate, and tauroursodeoxycholic acid in liver were elevated 35.94, 24.36, and 18.71-fold, respectively. PI(P-16:0/18:1(9Z)), PG(19:0/16:0), PS(P-16:0/20:2(11Z,14Z)), PI(22:1(11Z)/12:0), and PE(21:0/0:0) in cecum were enhanced 884, 640.96, 226.63, 210.10, 45.13-fold in comparison with the lean mice. These metabolites were the most important biomarkers for discriminating between the obese and lean mice. In addition, cecum contents metabolites were strongly correlated with hepatic metabolites through gut-liver axis analysis.

**Conclusions:**

HFD increased lipid profiles (i.e. glycerophospholipids, PC, PE, PI, PG, and PS) and total bile acid (primary and secondary bile acid) in liver and cecum, suggesting that they may play an important role in the progression of obesity. These metabolites can be used to better understand obesity and related disease induced by HFD. Furthermore, the level alterations of these metabolites can be used to assess the risk of obesity and the therapeutic effect of obesity management.

## Background

The obesity epidemic and its metabolic complications are a major public health problem around the world [1]. Obesity is a complex and multifactorial disorder, involving the imbalance between energy intake and expenditure, which leads to an excessive accumulation of lipids. Many studies have shown that the disorder of lipid metabolism is one of the main characteristics of obesity. Lipid metabolism disorders refer to abnormal lipid profile alterations in the blood, liver and other tissues, including hypercholesterolemia, hyperlipidaemia, hyperglycaemia, and non-alcoholic fatty liver syndrome (NAFLS) [2]. At present, lipid metabolism disorders is generally considered as the risk factor leading to low quality of life and increased burden on society. Many drugs are commonly used for obesity treatment. However, some side or toxic effects have limited their clinical application [3]. Therefore, the development of a safe and effective drug for the treatment of obesity has become an international research hotspot.

Gut microbiota is now considered to be an important factor affecting human health and disease [4]. Emerging evidence suggests a strong interaction between gut microbiota and liver, known as “gut-liver axis” [5]. These interactions occur through metabolites and begin at birth [6]. This relation has been based on the evidence that the liver represents the first line defense against gut-derived antigens and approximately 70% its blood supply comes from the portal vein, the direct venous outflow of the gut [7, 8]. The abundance of bacterial metabolites may have a significant impact on human physiology and the development of disease. Metabolites derived from the gut microbiota, such as lipopolysaccharides, bile acid derivatives, amino acid derivatives, and SCFAs, are important signaling molecules that link the gut microbiota to the host [9, 10]. High-fat diet may affect the host metabolism and modulate through manipulating the gut microbiota to increase hepatic injury in animal models. Due to the close anatomical and functional associations between the liver and gut, the dysfunction of the microbial ecosystem in obesity leads to disturbances of the gut barrier, which induce the direct delivery of gut-derived gut microbes and bacterial metabolites to the liver through the portal vein and induce immune response and aggravate liver injury [11].

Deciphering signatures specific to liver alterations would be most useful for future obesity diagnostic biomarkers. Several metabolites, including fatty acids, lysophophatidylcholines (lysoPCs), and branched-amino acids (BCAAs) have been identified as potential biomarkers of obesity and related diseases through human and animal studies [12], but understanding obesity metabolism based on a few identified metabolites is limited. More identification of obesity-related metabolites is necessary for our further understanding of obesity metabolism. Although many studies have reported the relationship between the gut microbiota and liver metabolites, very little has been reported about the relationship between metabolites derived from gut and liver metabolites in dietary-induced obesity. We herein study the gut microbially derived metabolite signatures associated with liver metabolite alternations, focusing on their relationship with obesity progression. We specifically focus on which microbial metabolites signatures are specific to liver injury. As an emerging discipline, metabolomics provide a powerful method for the discovery of biomarkers in biological systems [13]. At present, liquid chromatography–mass spectrometry (LC–MS) has many advantages, such as sensitivity and reproducibility, and is one of the commonly used techniques in metabolomics research [14]. We previously established the obesity model in mice induced by HFD, and the results revealed that HFD led to the increased levels of TG, TC, ALT and AST in serum and liver, and lipid droplets accumulation in liver [15]. However, the mechanism of obesity and the organic dysfunction associated with obesity is not clearly understood. To explore the potential characteristic metabolites that are associated with obesity, a nontargeted metabolomics technique is performed to discover potential cecal and liver metabolites and integrative analyses through gut-liver axis are applied to find relationship between the liver specific metabolites and cecal metabolites composition. By integrating metabolomics information, we would better understand the interactions between gut and host metabolism. On this basis, combined with the changes of liver and cecum contents metabolites, the common changes of the gut-liver axis were established, which provides basic data for understanding the influence of gut-derived metabolic process of obesity and its mechanism, while also providing a rationale for metabolites-based interventions against obesity.

## Materials and methods

### Animals and experimental design

Eighteen female 7-week-old Kunming mice were obtained from Vital River Laboratory Animal Technology Co. Ltd (Beijing, China), and housed in cages under a 12 h light–dark cycle. After 1-week acclimation period, the mice were randomly divided into two treatment groups (n = 9) and fed with (1) normal-chow diet, (2) high fat diet (HFD) composed of 67% (w/w) normal chow, 10% lard, 20% sucrose, 2.5% cholesterol, and 0.5% sodium cholate (Keao Xieli Feed Co., Ltd, Beijing, China) according to the previous report with slight modifications [16].

### Collection of liver and cecum contents samples

After 16 weeks of experimental administration, the mice were fasted 6 h. The blood samples were collected from eighteen mice and placed at room temperature for 1 h. Serum was obtained by centrifuged (3000*g*, 10 min), and stored at − 80 °C for further analysis. Liver tissues were washed with ice-bold buffered saline, weighed and homogenized in saline with a homogenizer. The homogenates were then centrifuged (4 °C, 3000*g*, 20 min) for further analysis. The Livers and cecum contents were extracted and snap-frozen in liquid nitrogen, and then stored at ‒ 80 °C for metabolomics analysis.

### Untargeted liver metabolomics analysis

The Liver tissues (60 mg) were homogenized, vortexed for 1 min and dissolved in 800 μL solvent mixture containing methanol/acetonitrile (1:1, v/v). The samples were vortexed for 1 min, and sonicated for 30 min on ice and the sonication cycle was repeated for twice, and then placed at − 20 °C for 1 h, and then centrifugation at 14,000*g* for 20 min at 4 °C. The supernatants were collected for ultraperformance liquid chromatography-quadrupole-time-of-flight mass spectrometry (UHPLC-Q-TOF/MS) analysis. In parallel to the preparation of the test samples prepare a bulk quality control (QC) sample, which is made by mixing equal volumes from each of the samples. The QC samples were used to monitor the LC–MS response.

Metabolic analysis of liver samples were carried out by an Agilent 1290 Infinity LC ultra-high pressure lipid chromatograph (UHPLC) system (Agilent, Santa-Clara, CA, USA) equipped with an electrospray ionization source operating in positive and negative ion mode. For the metabolomics analysis, an ACQUITY UPLC HSS T3 column (2.1 × 100 nm, 1.8 μm, Waters MS Technologies, Manchester, UK) was used. The column was maintained at 25 °C and eluted at a flow rate of 300 μL/min. The mobile phase was composed of A (25 mM ammonium acetate and 25 mM ammonium hydroxide in water) and B (acetonitrile). The process of linear gradient elution as follows: 0–1.0 min, 95% B; 1.0–14.0 min, 95–65% B; 14.0–16.0 min, 65–40% B; 16.0–18.0 min, 40% B; 19.0–18.1 min, 40–95% B; 18.1–23.0 min, 95% B. The auto sampler was maintained at 4 °C.

The mass spectrometer (MS) was a Triple TOFTM 5600 system (AB/Sciex, Foster City, USA) equipped with an electrospray (ESI) as ionization source in positive (ESI+) and negative (ESI−) ion mode. The MS properties were set as follows: scan range, *m*/*z* of 60–1000 Da; product ion scan *m*/*z* range, 25–1000 Da; TOF MS scan accumulation time 0.20 s/spectra; product ion scan accumulation time, 0.05 s/spectra; Ion Source Gas1(Gas1), 60 psi; Ion Source Gas2 (Gas2), 60 psi; Curtain gas (CUR): 30 psi; source temperature, 600 °C; Ion Sapary Voltage Floating (ISVF), ± 5500 V; Declustering potential (DP): ± 60 V; Collision energy, 35 ± 15 eV. MS/MS data were acquired in the information dependent acquisition (IDA) mode and using high sensitivity modes. The settings of IDA as follows: exclude isotopes within 4 Da, candidate ions to monitor per cycle, 6. Meanwhile the QC samples were analyzed to monitor the system stability and data quality.

### Untargeted cecum contents metabolomics analysis

50 mg of cecal sample was weighted to an EP tube, and 1000 μL extract solution (acetonitrile: methanol: water = 2: 2: 1) with 1 g/mL internal standard was added. After 30 s vortex, the samples were homogenized at 35 Hz for 4 min and sonicated for 5 min on ice. The homogenization and sonication cycle was repeated for 3 times. Then the samples were incubated for 1 h at − 40 °C and centrifuged at 12,000*g* for 15 min at 4 °C. The resulting supernatant was transferred to a fresh glass vial for analysis. The quality control (QC) sample was prepared by mixing an equal aliquot of the supernatants from all of the samples.

LC–MS/MS analyses were performed using an UHPLC system (1290, Agilent Technologies) with a UPLC HSS T3 column (2.1 mm × 100 mm, 1.8 μm) coupled to Q Exactive mass spectrometer (Orbitrap MS, Thermo). The mobile phase A was 0.1% formic acid in water for positive mode, and 5 mmol/L ammonium acetate in water for negative mode, and the mobile phase B was acetonitrile. The elution gradient was set as follows: 0–1.0 min, 1% B; 1.0–8.0 min, 1–99% B; 8.0–10.0 min, 99% B; 10.0–10.1 min, 99% ~ 1% B; 10.1–12 min, 1% B. The flow rate was 500 μL/min. The injected volume was 2 μL. The QE mass spectrometer was used for its ability to acquire MS/MS spectra on information-dependent acquisition (IDA) mode in the control of the acquisition software (Xcalibur 4.0.27, Thermo). In this mode, the acquisition software continuously evaluates the full scan MS spectrum. The ESI source conditions were set as following: sheath gas flow rate as 45 Arb, Aux gas flow rate as 15 Arb, capillary temperature 400 °C, full MS resolution as 70,000, MS/MS resolution as 17,500, collision energy as 20/40/60 in NCE mode, spray Voltage as 4.0 kV (positive) or − 3.6 kV (negative), respectively.

### Data processing

The raw UHPLC-Q-TOF/MS data were converted to the mzXML format using ProteoWizard and processed with an in-house program, which was developed using R and based on XCMS, for peak detection, extraction, alignment, and integration. Then an in-house MS2 database (BiotreeDB) was applied in metabolite annotation. The cutoff for annotation was set at 0.3.

### Statistical analysis

The final dataset was imported to SIMCA15.0.2 software package (Umetrics, Umeå, Sweden) for multivariate statistical analysis. Orthogonal partial least squares discriminant analyses (OPLS-DA) was performed to distinguish the overall differences in metabolic profiles between obese and lean samples and to find differential metabolites between the two groups. Then, a sevenfold cross validation was performed to calculate the value of R2 and Q2. R2 indicates how well the variation of a variable is explained and Q2 means how well a variable could be predicted. To check the robustness and predictive ability of the OPLS-DA model, 200 times permutations was further conducted. Afterward, the R2 and Q2 intercept values were obtained. Furthermore, the value of variable importance in the projection (VIP) of the first principal component in OPLS-DA analysis was obtained. It summarizes the contribution of each variable to the model. The metabolites with VIP > 1 and *p* < 0.05 (student *t* test) were considered as significantly changed metabolites. The Kyoto Encyclopedia of Genes and Genomes (KEGG) database was used to analyze the differential metabolites. In addition, all differentially abundant metabolites were queried against KEGG (http://www.kegg.jp/) and mapped to KEGG pathways. Enrichment analysis was performed to further explore the impact of differentially expressed metabolites and to analyze the internal relationships between differentially expressed metabolites using the Fisher’s exact test. Only functional categories and pathways with *p* < 0.05 were considered to have significant enrichment.

## Results

### Metabolic findings in liver samples

**Fig. 1:**
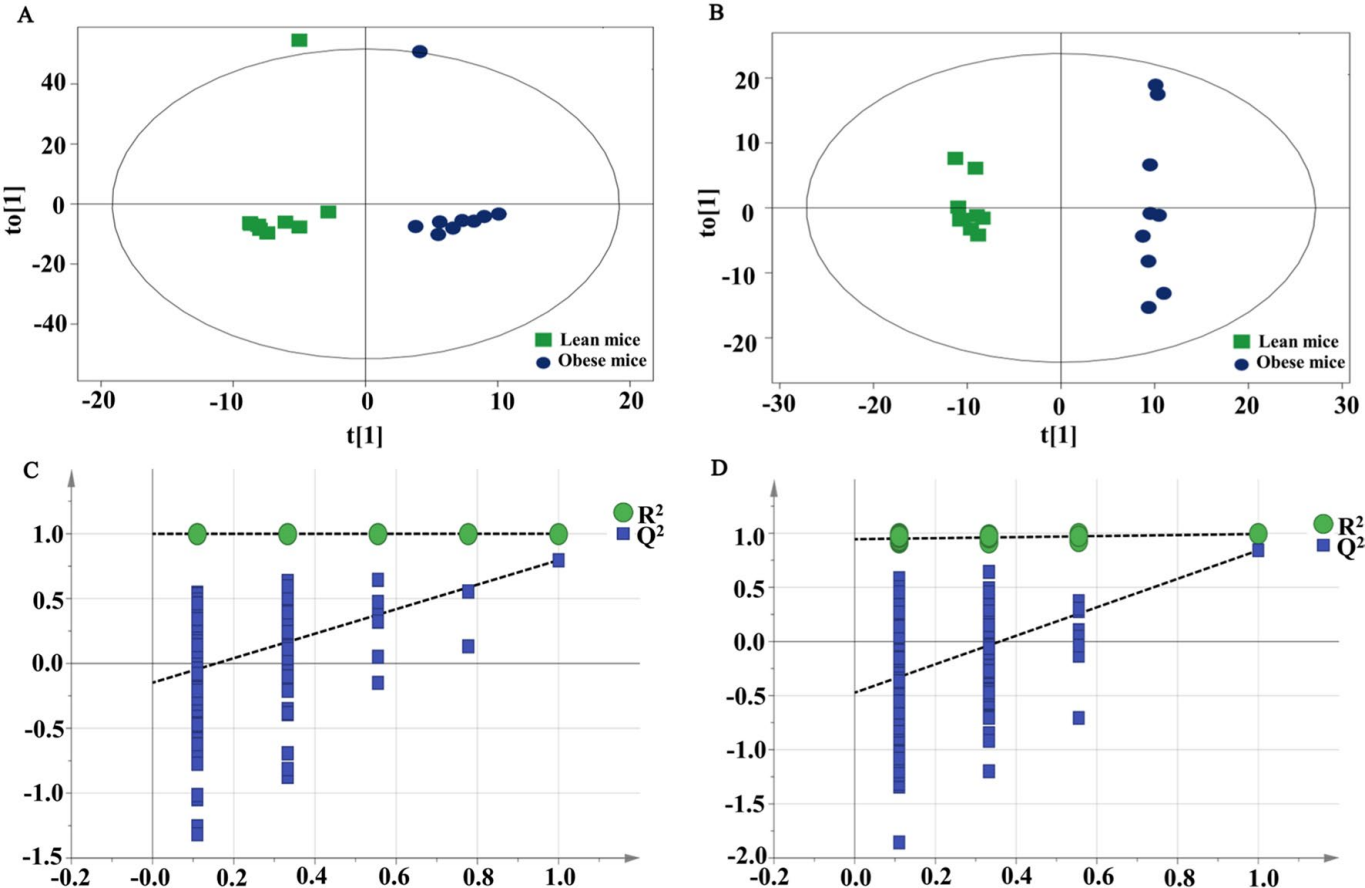
Multivariate data analyses of hepatic metabolites based on UPLC-Q-TOF/MS analysis. **A**, **B** Score scatter plot of OPLS-DA model for the obese versus lean mice in positive and negative modes. **C**, **D** Permutation test of OPLS-DA model for group obese versus lean mice

The hepatocytes are most exposed to gut-derived toxic factors through gut-liver axis, including bacteria and bacterial products. Examination of the metabolites composition in the liver will provide more accurate understanding about liver lipid metabolism and signaling. UHPLC-Q-TOF MS liver metabolomics analysis discovered the different metabolomics features in the obese and lean groups. As shown in Fig. 1A, B, OPLS-DA score plots displayed good separation effect between the obese and lean mice, indicating that high-fat diet induced significant systemic changes and obesity was successfully induced. The parameters of OPLS-DA models including R^2^X = 0.808, R^2^Y = 1, Q^2^ = 0.796 for positive data, and R^2^X = 0.572, R^2^Y = 0.993, Q^2^ = 0.842 for negative data were obtained. The permutation test was used to guard against overfitting of the OPLS-DA models. As shown in Fig. 1C, D, validation with 200 random permutation tests generated intercepts R^2^ = 0.999 and Q^2^ = − 0.148 for positive data, and R^2^ = 0.944 and Q^2^ = − 0.473 for negative data, which demonstrated that the OPLS-DA models were robust and reliable without overfitting.

**Fig. 2:**
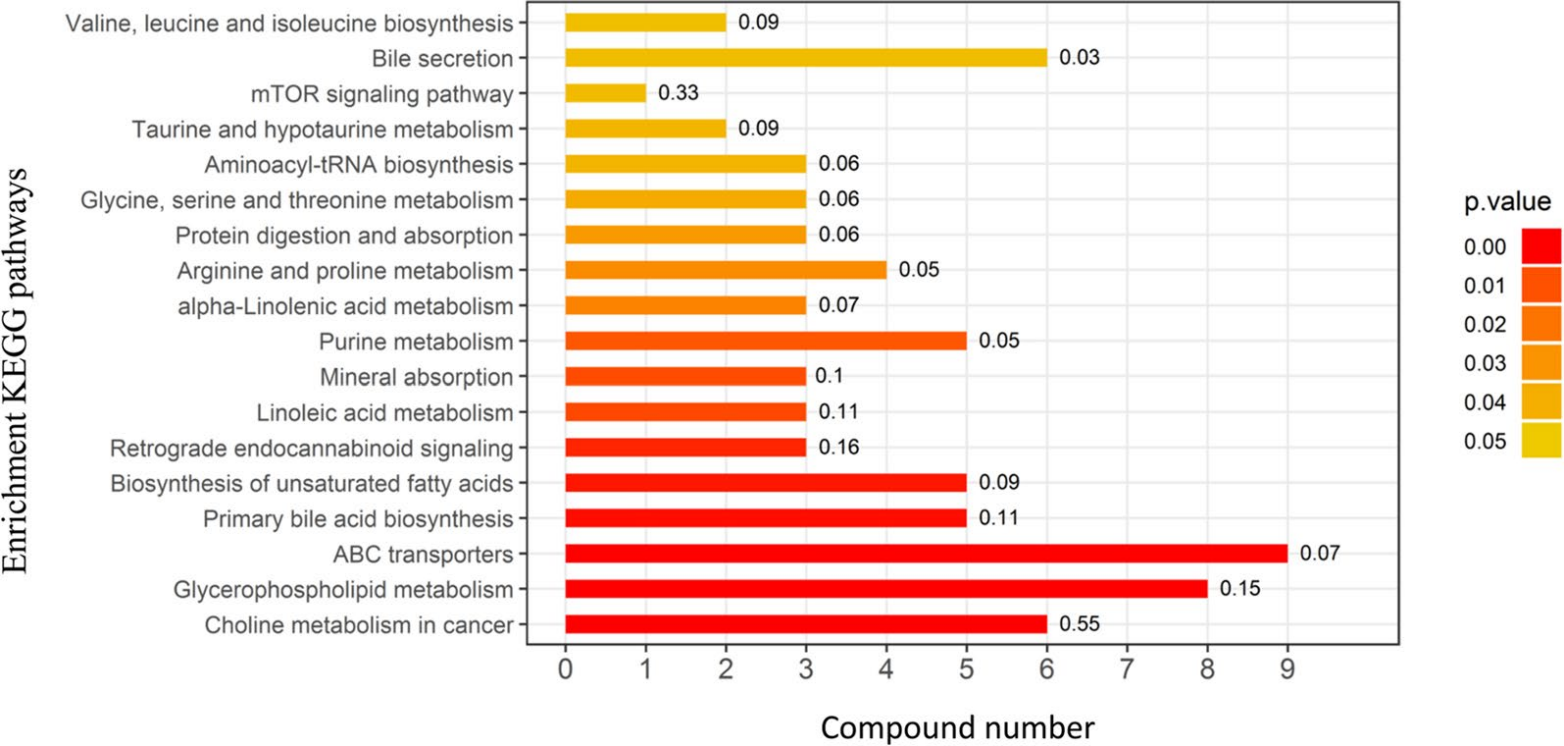
Enrichment analysis results of KEGG pathway between obese and lean mice

The significantly differential metabolites were selected based on the criteria of an OPLS-DA model VIP > 1 and *p* < 0.05. UHPLC-Q-TOF MS metabolomics analysis dis-covered 19 differentially enriched metabolites between the obese and lean groups. Twenty-six of the metabolites were elevated in the obese group (fold change > 1.2) while Twenty-eight of them were decreased (fold change < 0.7). As shown in Fig. 2, according to the KEGG reference pathways, indicators with significantly discriminative power were the “ABC transporters”, “Glycerophospholipid metabolism”, “Choline metabolism in cancer”, “Primary bile acid biosynthesis”, and “Biosynthesis of unsaturated fatty acid”, which showed significant enrichment with HFD treatment. As shown in Table 1, the levels of choline, cytidine 5′-diphosphocholine (CDP-choline), phosphorylcholine, O-phosphoethanolamine, sn-glycerol 3-phosphoethanolaminesn, and glycerophosphocholine involving in glycerophospholipid metabolism pathway, were significantly increased with fold changes of 1.38, 2.13, 2.39, 1.71, 1.94, and 2.13 respectively (*p* < 0.05), while PC (16:0/16:0) and 1-stearoyl-2-oleoyl-sn-glycerol 3-phosphocholine were significantly decreased with fold changes of 0.46 and 0.6, respectively (*p* < 0.05), which indicates high relevance to the difference between normal and high-fat diet fed mice. Compared to the lean group, HFD intervention significantly increased the amounts of oleic acid (3.89-fold), while the levels of eicosapentaenoic acid, (4Z,7Z,10Z,13Z,16Z,19Z)-4,7,10,13,16,19-docosahexaenoic acid, and alpha-linolenic acid involving in biosynthesis of unsaturated fatty acid pathway, were significantly decreased with fold changes of 0.30, 0.62, and 0.67 (*p* < 0.05), respectively.

**Table 1:** Potential biomarkers in liver based on the UPLC-Q-TOF/MS analysis between the obese and lean mice

Ionization mode	RT(s)	*m*/*z*	Metabolites	Metabolic pathway	VIP	Fold change	*p* value
ESI (+)	788.63	198.0517674	sn-Glycerol 3-Phosphoethanolaminesn	Glycerophospholipid metabolism	1.95	1.94	0.001
ESI (+)	780.035	104.1058352	Choline	Glycerophospholipid metabolism	1.29	1.38	0.0009
ESI (+)	878.204	489.1135327	Gytidine 5’-diphosphocholine	Glycerophospholipid metabolism	1.84	2.13	0.032
ESI (+)	915.7575	142.0254256	O-Phosphoethanolamine	Glycerophospholipid metabolism	1.02	1.71	0.002
ESI (+)	862.561	258.1094388	Glycerophosphocholine	Glycerophospholipid metabolism	1.45	2.13	0.014
ESI (+)	315.6605	756.5520133	PC(16:0/16:0)	Glycerophospholipid metabolism	1.91	0.46	0.013
ESI (−)	717.05	242.0794821	Phosphorylcholine	Glycerophospholipid metabolism	1.62	2.39	0.012
ESI (+)	363.218	285.22	Eicosapentaenoic acid	Biosynthesis of unsaturated fatty acids	2.00	0.30	0.011
ESI (−)	69.324	327.2320636	(4Z,7Z,10Z,13Z,16Z,19Z)-4,7,10,13,1 6,19-Docosahexaenoic acid	Biosynthesis of unsaturated fatty acids	12.62	0.62	0.0058
ESI (−)	356.1175	281.2478597	Oleic acid	Biosynthesis of unsaturated fatty acids	4.70	3.89	0.029
ESI (−)	73.247	277.2171703	Alpha-linolenic acid	Biosynthesis of unsaturated fatty acids	5.06	0.67	0.039
ESI (−)	302.251	391.2844083	Chenodeoxycholic acid	Primary bile acid biosynthesis	6.08	35.94	2.9E−05
ESI (+)	413.101	516.2991879	Taurocholic acid	Primary bile acid biosynthesis	1.80	0.35	0.034
ESI (+)	592.106	126.0208716	Taurine	Primary bile acid biosynthesis	10.12	0.41	0.002
ESI (−)	267.2065	498.2881509	Taurochenodeoxycholic acid	Primary bile acid biosynthesis	21.0	24.36	0.005
ESI (−)	351.388	407.2790416	Cholic acid	Primary bile acid biosynthesis	2.96	0.62	0.035
ESI (+)	316.61	517.3294166	Taurodeoxycholic acid	–	1.37	5.88	0.004
ESI (+)	326.4925	482.2922443	Tauroursodeoxycholic acid	–	4.31	18.71	0.011
ESI (−)	73.91	482.2933108	Taurolithocholic acid	–	1.60	4.36	0.003

*ESI+* electrospray ionization in positive ion mode, *ESI−* electrospray ionization in negative ion mode, *RT* retention time, *VIP* variance importance for projection, *PC* phosphatidylcholine

Changes in circulating bile acids are associated with obesity and related diseases. In mice, more than 95% conjugated bile acids are taurine-conjugated bile acids, while glycine-conjugated bile acids are more abundant in human [17]. Consistent with the above report, most of bile acids in the liver were taurine-conjugated in this study. The levels of chenodeoxycholic acid (CDCA), Taurochenodeoxycholic acid (TCDCA) and tauroursodeoxycholic acid (TUDCA) in primary bile acid biosynthesis pathway were increased with the fold changes of 35.94, 24.36 and 8.71 (*p* < 0.05), respectively, while the levels of cholic acid (CA), taurocholic acid (TCA) and taurine were decreased with fold changes of 0.62, 0.35, and 0.41 (*p* < 0.05), respectively, which were potential markers related to obesity. Additionally, HFD intervention significantly increased taurodeoxycholic acid (TDCA) and taurolithocholic acid (TLCA) belonging to secondary bile acid with the fold changes of 5.88 and 4.36 (*p* < 0.05), respectively. Besides on these results, we observed that several metabolites were also significantly changed, including the levels of purines, nicotinamide, and amino acids (data not shown).

### Metabolic findings in cecum contents samples

**Fig. 3:**
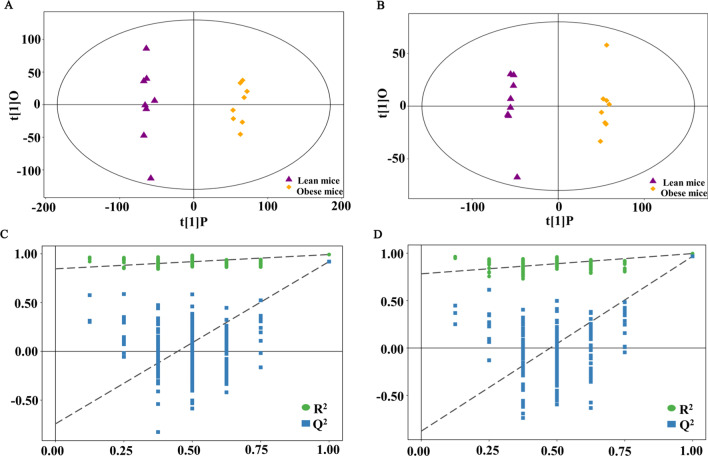
Score plots and permutation test derived from the cecum contents of mice based on UPLC-Q-TOF/MS analysis. **A**, **B** Score scatter plot of OPLS-DA model for the obese versus lean mice in positive and negative modes. **C**, **D** Permutation test of OPLS-DA model for group obese versus lean mice

Gut-derived microbial products can reach the liver by gut-liver axis, and then have an effect on liver metabolism. In the present study, a metabolomics study of cecum contents based on UHPLC-Q-TOF/MS was conducted in mice fed with HFD. Cecum contents metabolomics presented similar results with that of liver tissue. As shown in Fig. 3A, B, OPLS-DA score plots and cluster analysis from both of positive and negative modes showed altered patterns with distinct differences in obese group versus the lean group, indicating that high-fat diet treatment induced the negative effects. The parameters of OPLS-DA models including R^2^X = 0.384, R^2^Y = 0.992, Q2 = 0.92 for positive data, and R^2^X = 0.409, R^2^Y = 0.996, Q^2^ = 0.967 for negative data were obtained. In addition, the OPLS-DA models validated by the permutation test with 200 random permutation tests generated intercepts R^2^ = 0.85 and Q^2^ = − 0.74 for positive data, and R^2^ = 0.78 and Q^2^ = -0.88 for negative data (Fig. 3C, D), which demonstrated that the goodness of fit of the data.

As shown in Table 2, compared to the lean mice, it can be seen that the 43 characteristic metabolites with VIP > 1 and *p* < 0.05 belong to glycerophospholipids, bile acids, the fatty acyl group (5-acetamidovalerate, 11-dehydro-thromboxane B2, resolvin D5, alpha-dimorphecolic acid, 2,3-dinor-6-keto-prostaglandin F1 a, hexadecanedioic acid mono-L-carnitine ester, ethyl oleate, docosapentaenoic acid (22n-3), 9,10-epoxyoctadecanoic acid, 13-OxoODE), and glycerolipids (MG(0:0/18:3(9Z,12Z,15Z)/0:0), gingerglycolipid A) were filtered and identified as potential biomarkers. Twenty-three of the metabolites involving in glycerophospholipid metabolism were significantly changed (*p* < 0.05), twenty of which were elevated, while three metabolites were reduced after HFD intervention. Nine phosphatidylcholines (PC) species, including PC(0:0/14:0), PC(O-16:0/2:0), PC(2:0/O-16:0)[U], PC(7:0/8:0)[U], PC(O-15:0/0:0), PC(O-16:1(11Z)/2:0), PC(O-8:0/O-8:0), PC(8:0/7:0)[U], and PC(8:0/6:0), were significantly increased 18.18, 17.86,17.04, 9.20, 6.40, 4.15, 2.77, 1.56, and 1.52-fold, respectively, which contributed to evaluate the difference between normal and high-fat diet mice. In addition, the levels of PG(19:0/16:0), PS(P-16:0/20:2(11Z,14Z)), PI(P-16:0/18:1(9Z)), PI(22:1(11Z)/12:0), PE (21:0/0:0), and PE (13:0/0:0) were increased 640.96, 226.63, 884.00, 210.10, 45.13, and 8.17-fold with VIP > 1.5, respectively, which were the most important hepatic metabolites for discriminating between lean and obese mice. However, the levels of LysoPC(18:1(9Z)), LysoPE(15:0/0:0), and LysoPE(0:0/20:3(11Z,14Z,17Z)) were negatively affected with 0.32, 0.44, and 0.30-fold, respectively. L-palmitoylcarnitine involved in fatty acid metabolism was enhanced 16.16-fold. The levels of three metabolites involving in biosynthesis of unsaturated fatty acid, including adrenic acid, icosenoic acid, and stearic acid were elevated 1.39, 2.31, and 1.44-fold, respectively.

**Table 2:** Potential biomarkers in cecum contents based on the UPLC-Q-TOF/MS between obese and lean mice

Ionization mode	RT(s)	*m*/*z*	Metabolites	Metabolic pathway	VIP	Fold change	*p* value
ESI (+)	366.533	523.3816793	PC(2:0/O-16:0)[U]	Glycerophospholipid metabolism	1.53	17.04	0.017
ESI (+)	330.811	524.3154739	PC(O-16:0/2:0)	Glycerophospholipid metabolism	1.99	17.86	2.82E−05
ESI (+)	314.942	495.3143924	PC(8:0/7:0)[U]	Glycerophospholipid metabolism	1.41	1.56	0.0051
ESI (+)	368.531	206.190139	PC(O-16:1(11Z)/2:0)	Glycerophospholipid metabolism	1.89	4.15	0.021
ESI (+)	464.138	522.354753	PC(7:0/8:0)[U]	Glycerophospholipid metabolism	1.49	9.20	0.001
ESI (+)	314.989	467.3192207	PC(0:0/14:0)	Glycerophospholipid metabolism	1.73	18.18	0.019
ESI (+)	319.178	481.3111877	PC(8:0/6:0)	Glycerophospholipid metabolism	1.05	1.52	0.040
ESI (+)	469.048	482.3602632	PC(O-8:0/O-8:0)	Glycerophospholipid metabolism	1.72	2.77	0.0013
ESI (+)	512.919	467.7871501	PC(O-15:0/0:0)	Glycerophospholipid metabolism	1.38	6.40	0.0078
ESI (−)	458.095	478.2949417	LysoPC(18:1(9Z))	Glycerophospholipid metabolism	1.39	0.32	0.009
ESI (+)	421.504	451.2983584	LysoPE(16:1(9Z)/0:0)	Glycerophospholipid metabolism	1.52	2.41	0.012
ESI (+)	477.56	510.3550402	LysoPE(0:0/20:0)	Glycerophospholipid metabolism	1.58	1.87	0.00042
ESI (+)	420.079	440.2764659	LysoPE(15:0/0:0)	Glycerophospholipid metabolism	1.62	0.44	0.0020
ESI (+)	399.0275	503.3580866	LysoPE(0:0/20:3(11Z,14Z,17Z))	Glycerophospholipid metabolism	1.34	0.30	0.030
ESI (+)	479.118	507.3640197	LysoPE(20:1(11Z)/0:0)	Glycerophospholipid metabolism	1.31	1.67	0.0066
ESI (+)	451.64	546.3549278	1-(8Z,11Z,14Z-eicosatrienoyl)-sn-glycero-3-phosphocholine	Glycerophospholipid metabolism	1.91	13.14	0.0027
ESI (+)	441.3365	360.2526586	PG(16:0/0:0)[U]	Glycerophospholipid metabolism	1.59	4.42	0.022
ESI (+)	541.67	765.5648852	PG(19:0/16:0)	Glycerophospholipid metabolism	1.96	640.96	0.00028
ESI (+)	506.869	524.4297664	PE(21:0/0:0)	Glycerophospholipid metabolism	1.90	45.13	0.0016
ESI (+)	393.4455	411.2499395	PE(13:0/0:0)	Glycerophospholipid metabolism	1.79	8.17	0.0029
ESI (+)	343.046	464.3364698	PS(P-16:0/20:2(11Z,14Z))	Glycerophospholipid metabolism	2.01	226.63	0.023
ESI (+)	519.713	821.5524549	PI(P-16:0/18:1(9Z))	Glycerophospholipid metabolism	2.03	884.00	0.00014
ESI (+)	439.284	837.547032	PI(22:1(11Z)/12:0)	Glycerophospholipid metabolism	2.01	210.10	1.34E−06
ESI (−)	545.547	331.264861	Adrenic acid	Biosynthesis of unsaturated fatty acid	1.04	1.39	0.027
ESI (−)	560.467	309.271544	Icosenoic acid	Biosynthesis of unsaturated fatty acid	1.35	2.32	0.00036
ESI (−)	639.652	283.264859	Stearic acid	Biosynthesis of unsaturated fatty acid	1.10	1.44	0.035
ESI (+)	421.994	400.2801629	L-Palmitoylcarnitine	Fatty acid metabolism	1.68	16.16	0.0025
ESI (+)	392.1765	500.2850825	Taurochenodesoxycholic acid	Primary bile acid biosynthesis	1.88	13.71	4.89E−06
ESI (−)	297.733	407.0633335	Cholic acid	Primary bile acid biosynthesis	1.41	4.55	0.0021
ESI (+)	394.4385	374.2895602	Ursodeoxycholic acid	Primary bile acid biosynthesis	1.67	0.33	8.58E−05
ESI (+)	363.583	483.332665	Lithocholyltaurine	–	1.49	13.57	0.030
ESI (+)	366.023	328.1413614	5-Acetamidovalerate	–	1.31	2.19	0.0056
ESI (+)	305.9705	368.2426992	11-Dehydro-thromboxane B2	–	1.74	0.25	1.01E−05
ESI (+)	441.3365	360.2526586	Resolvin D5	–	1.56	0.24	0.012
ESI (+)	532.899	279.1005666	Alpha-dimorphecolic acid	–	1.41	1.80	0.00058
ESI (+)	244.632	309.1805271	2,3-Dinor-6-keto-prostaglandin F1 a	–	1.16	0.64	0.030
ESI (+)	376.4805	430.263936	Hexadecanedioic acid mono-L-carnitine ester	–	1.87	9.49	0.00026
ESI (−)	482.271	303.2335177	Ethyl oleate	–	1.56	4.08	0.0022
ESI (−)	520.4375	329.2491524	Docosapentaenoic acid (22n-3)	–	1.23	2.07	0.011
ESI (−)	453.562	297.1071611	9,10-epoxyoctadecanoic acid	–	1.22	3.16	0.018
ESI (−)	558.79	700.557389	13-OxoODE	–	1.14	0.062	0.045
ESI (+)	411.144	352.2841858	MG(0:0/18:3(9Z,12Z,15Z)/0:0)	–	1.72	3.30	0.00039
ESI (+)	449.367	676.4258482	Gingerglycolipid A	–	1.63	0.034	0.0023

*ESI+* electrospray ionization in positive ion mode, *ESI−* electrospray ionization in negative ion mode, *LysoPC* lysophosphatidylcholine, *PC* phosphatidylcholine, *PE* phosphoethanolamine, *PG* phosphatidylglycerol, *PI* phosphatidyl inositol, *PS* phosphatidylserine, *RT* retention time, *VIP* variance importance for projection

Total bile acid metabolites were enhanced in comparison with the lean mice. The increased concentration of bile acid in obese mice might be associated with the disorder of liver function. As shown in Table 2, CA and TCDCA involving in primary bile acid biosynthesis were increased 4.55 and 13.71-fold, respectively. In addition, lithocholyltaurine was elevated 13.57-fold, while ursodeoxycholic acid (UDCA) was decreased 0.33-fold. When compared with the normal lean mice, the results of the total metabolites belong to the fatty acyls were also increased. The KEGG results revealed that glycerophospholipid metabolism, biosynthesis of unsaturated fatty acid, and primary bile acid biosynthesis in cecum contents were highly disturbed pathways by HFD.

## Discussion

**Fig. 4:**
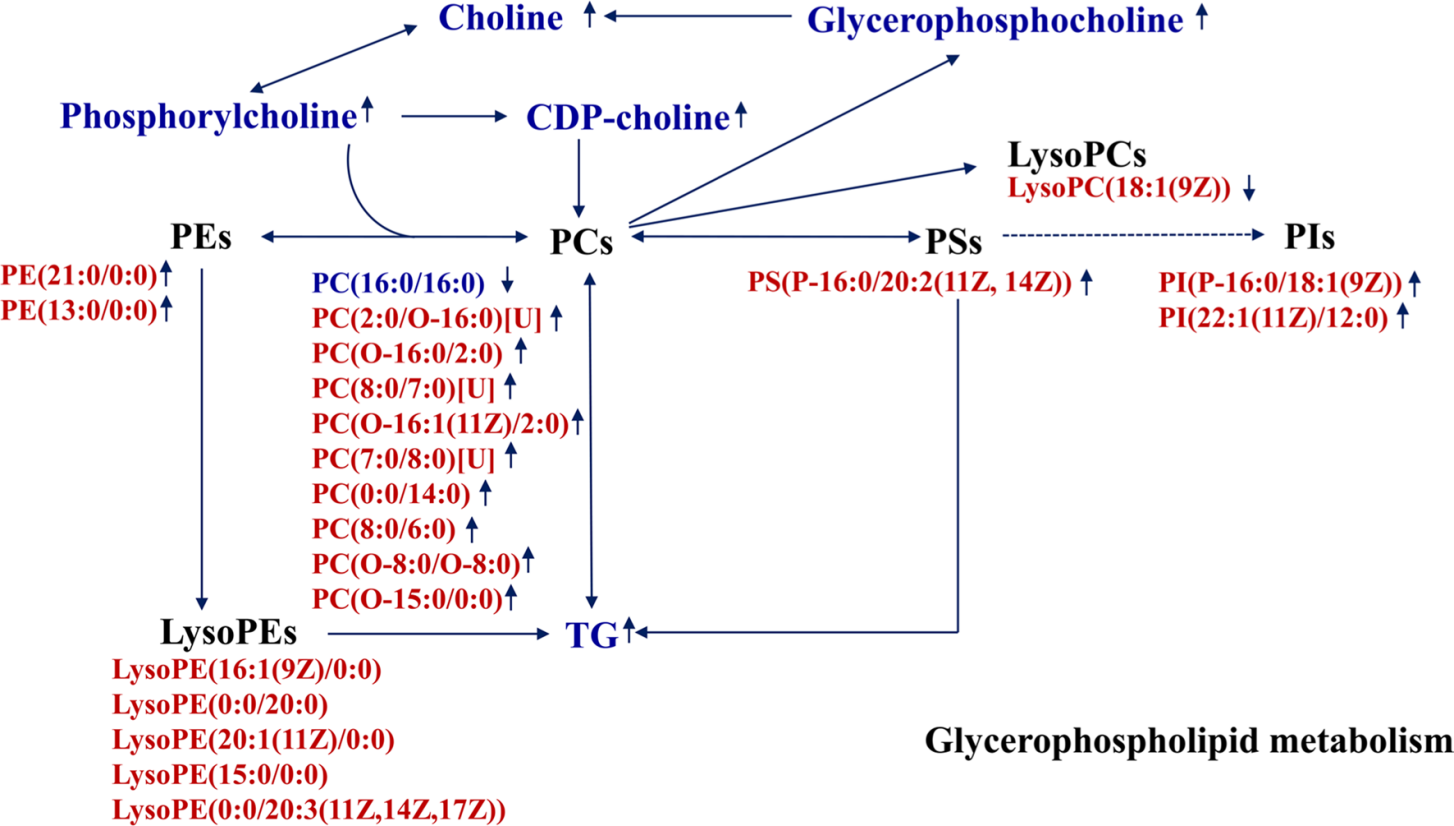
Glycerophospholipid metabolism pathway disturbed in the obese mice induced by a high-fat diet based on the metabolites of liver and cecum contents analysis. Metabolites names in blue or red indicated they were found in liver or cecum contents, respectively. After HFD intake, increased trend was indicated by ↑ and decreased trend was indicated by ↓. *CDP-choline* cytidine 5′-diphosphocholine, *LysoPC* lysophophatidylcholine, *LysoPE* lysophosphatidylethanolamine, *PC* phosphatidylcholine, *PE* phosphoethanolamine, *PI* phosphatidyl inositol, *PS* phosphatidylserine, *TG* triglyceride

**Fig. 5:**
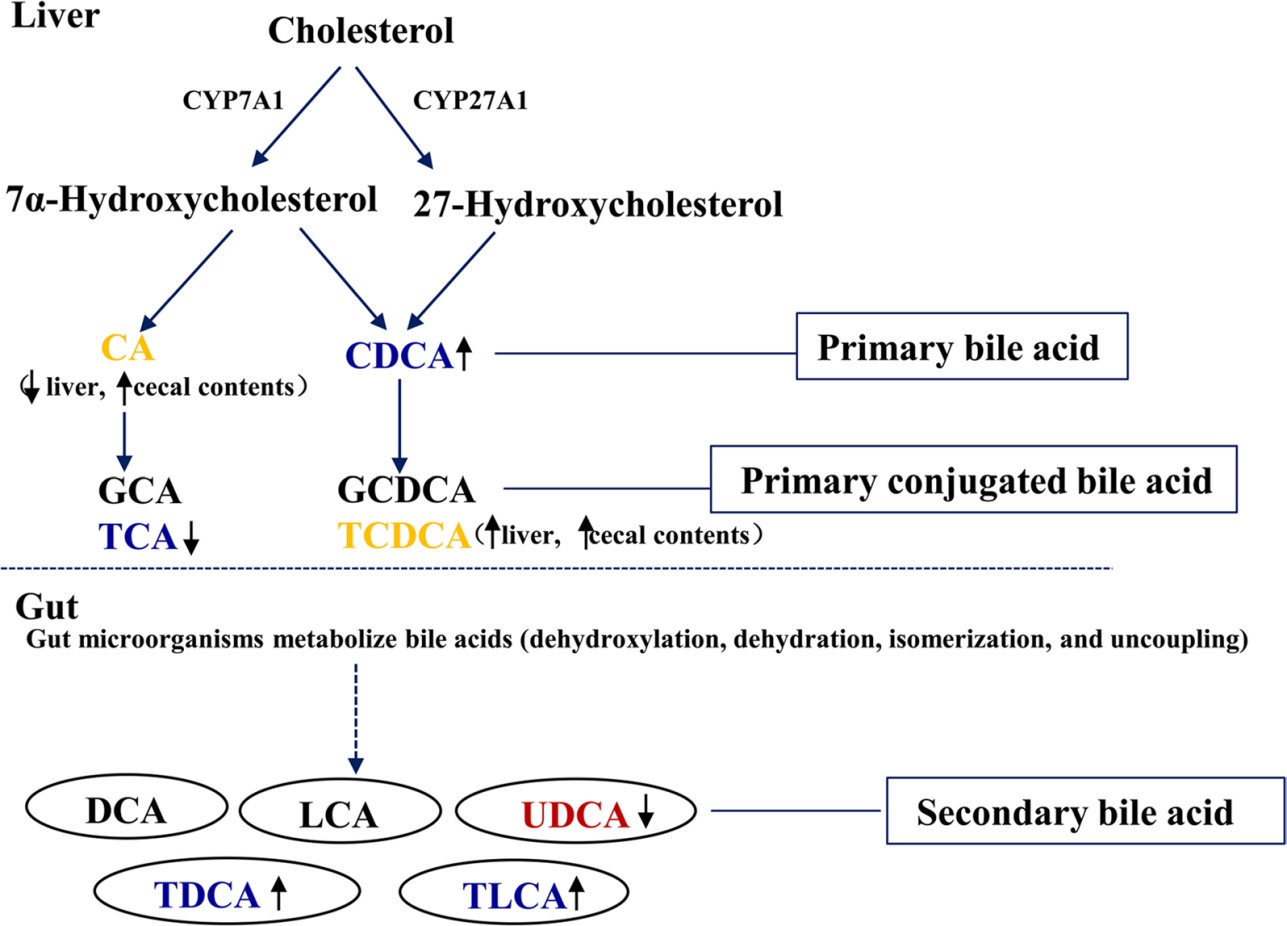
Bile acids metabolism pathway disturbed in the obese mice induced by a high-fat diet based on the metabolites of liver and cecum contents analysis. Metabolites names in blue or red indicated they were found in liver or cecum contents, respectively, and the metabolites in orange were found in both. The increased trend was indicated by ↑ and decreased trend was indicated by ↓. *CA* cholic acid, *CDCA* chenodeoxycholic acid, *TCA* taurocholic acid, *TCDCA* taurochenodeoxycholic acid, *TDCA* taurodeoxycholic acid, *TLCA* taurolithocholic acid, *UDCA* ursodeoxycholic acid

The Metabolites produced by the gut microbiota play important roles in mediating the complex interaction between the gut microbiota and the human holobiont [9]. In our previous study, HFD intake induced the disorders of liver and serum lipid, and alterations of gut microbiota composition in obese mice model fed with HFD [15]. In this study, we investigated hepatic metabolites and cecum contents of high-fat diet-induced obese mice using UHPLC-Q-TOF MS, and their metabolic profiles were compared with that of normal lean mice by multivariate statistical analysis. We found that some hepatic and cecum contents metabolites associated with lipid metabolism and obesity-related diseases were altered by HFD intake, and these changes in metabolic profiles helped distinguish between the obese and lean mice. Based on the determination of the identified metabolites, the metabolic pathways and gut-liver axis analysis of obesity induced by a high-fat diet were proposed, which were shown in Figs. 4 and 5. Some of these metabolites have been well studied, whereas others have not.

Disorders of glycerophospholipid and fatty acid metabolism have been found to be directly related to the initiation and development of hyperlipidemia. In hepatic metabolites, compared to the lean mice, the overall levels of glycerophospholipids were increased, indicating that HFD led to lipid disorders. The levels of most glycerophospholipids were enhanced in the liver of obese mice, including choline, sn-glycerol phosphoethanolaminesn, CDP-choline, O-phosphoethanolamine, glycerophosphocholine, and phosphorylcholine. Excess glycerophospholipids were positively associated with fat accumulation, which agrees with the finding that an increased glycerophospholipid metabolites level in obese mice was positively correlated with fat accumulation [12]. Choline, like phospholipids, is an essential nutrient for the maintenance of normal liver function [18], which is absorbed mainly by the small intestine and completely metabolized in the liver [19]. The finding of the increased choline in liver was consistent with the observation that more choline is correlated with greater risks of having hepatic steatosis, NASH, and lobular inflammation [20]. A research also showed that plasma levels of free choline are positively correlated with the grade of liver steatosis and fibrosis [21]. Our results were inconsistent with a previous study in which choline-related metabolites such as choline, phosphatidylcholine, and glycerol 3-phosphate were elevated in HFD-fed mice [22]. A previous research showed that choline intake was inversely associated with the risk of nonalcoholic fatty liver disease (NAFLD) [23]. PCs are mainly synthesized through the cytidine diphosphate (CDP)-choline pathway and the PE N-methyltransferase (PEMT) pathway. However, PC (16:0/16:0) in the liver was negatively affected. This is in harmony with previous studies that reduced PC biosynthesis impairs the secretion of VLDL in the liver, thus leading to the pathogenesis of hepatic steatosis [24, 25]. The lipidomic analysis of the liver of NAFLD patients demonstrated increased TG levels and decreased PC levels [26]. Several researches also showed that PC treatment could alleviate fat accumulation [27, 28].

We found that there was a possible link between the altered microbial metabolites and liver metabolites. Most of glycerophospholipid metabolites were also enhanced in cecum contents. In addition to PC species, we also found many other glycerophospholipids, such as phosphoethanolamine (PE), phosphatidyl inositol (PI), and phosphatidylserine (PS) species, were significantly increased in the HFD group. These findings are in accordance with the notion that glycerophospholipids play several important roles in the development of NAFLD [29, 30]. Nine PCs increased, which is in accordance with a research that an enhanced PC level in plasma was positively correlated with fat accumulation [12]. In addition, excessive PCs in the cecum may be explained by PC synthesized in the liver can be secreted into bile. Biliary PC is hydrolyzed by phospholipases to lysoPC and fatty acid, which are then absorbed through the intestinal brush border membrane [31]. On the other hand, PC produced in enterocytes is secreted into the intestinal lumen, forming part of the hydrophobic mucus layer [32]. A decrease in the PC content in the mucus layer is a characteristic of ulcerative colitis, a chronic inflammatory condition of the terminal ileum and colon [32]. These results indicated that the gut attempts to maintain phospholipid homeostasis and cellular integrity through increasing the content of PC. LysoPC resulted from the partial hydrolysis of phosphatidylcholines (PC), removing one of the fatty acid groups. Changes in LysoPCs levels are thought to be correlated with diseases with abnormal energy status, such as obesity and hyperlipidemia [33]. In contrast to the lean mice, the level of LysoPC(18:1(9Z)) was decreased after HFD intervention. This is not consistent with a study that LysoPC(18:1(9Z)) was enhanced in rats on a high-fat diet [34]. LysoPC11(18:1(9Z)), LysoPE (15:0/0:0), and LysoPE (0:0/20:3(11z, 14z, 17z)) were decreased, while lysoPE(16:1(9Z)/0:0), LysoPE(0:0/20:0), and LysoPE(20:1(11Z)/0:0) were enhanced in obese mice, which were partly matched with other reports that eleven LysoPCs and 2 lysoPEs were reduced in serum in the mice fed with HFD [12]. Furthermore, PI(P-16:0/18:1(9Z)), PI(22:1(11Z)/12:0), PE(21:0/0:0), PE(13:0/0:0), and PS(P-16:0/20:2(11Z,14Z)) were significantly enhanced after HFD intake. A previous report showed that the levels of most of PC, PE, PI, and PS species were significantly decreased in liver of NAFLD rats [35]. PE(21:0/0:0) and PE(13:0/0:0) are two kinds of glycerophosphoethanolamines. Phosphoethanolamine (PE) is a class of phospholipids found in biological membranes. Together with PC, PS, PI, PE represents the backbone of most biological membranes. Phosphoethanolamines in food break down to form phosphoethanolamine-linked amadori products, which act as a part of the Maillard reaction [36]. These products accelerate membrane lipid peroxidation and cause oxidative stress on cells exposed to them [37]. Phosphatidylserine (PS) and phosphatidylglycerol (PG) are endogenous phospholipids with assumed anti-inflammatory potential. PG is a glycerophospholipid, generally considered to be a precursor to the synthesis of cardiolipin (CL), a mitochondrial signature phospholipid that is necessary for dynamic mitochondrial function [38]. Lack of PG in mammalian cells results in CL deficiency, mitochondrial dysfunction, and decreased adenosine triphosphate production [39]. Interestingly, PS (P-16:0/20:2(11Z, 14Z)), PG(19:0/16:0), and PG(16:0/06:0) [U] were significantly increased 226.63, 640.96, and 4.42-fold, respectively, which were important biomarkers in cecum. These results can be explained by that the gut attempts to maintain phospholipid homeostasis by increasing the content of PS and PG. These findings may indicate that the specific functions of different glycerophospholipids still require further clarification. The relationship between the differential hepatic and cecum contents metabolites in glycerophospholipid metabolism summarized shown in Fig. 4. Further studies are required to correlate obesity with glycerophospholipids. Consistently, pathway analysis results showed that glycerophospholipid metabolism was closely involved in hepatic and cecal metabolites metabolism.

There exists highly efficient bile acid preservation and recycling system within the body, which is termed the enterohepatic circulation. Bile acids, which are biosynthesized by the catabolism of cholesterol in the liver, are involved in regulating lipid absorption and metabolism [40, 41], and helping maintain the homeostasis of glucose, cholesterol, and triglyceride in the liver. In addition, bile acids, acting as a detergent for cellular membranes, can directly affect gut microbiota by causing membrane disruption through their amphipathic properties. The deconjugation of bile salt hydrolase (BSH) produced by gut microbiota is a prerequisite for the downstream modifications by 7-alpha-dehydroxylase to produce DCA and LCA, or by 7-alpha-hydroxysteroid dehydrogenase (HSDH) to produce UDCA. Gut microbiota convert primary bile acids to secondary bile acids, which has an important effect on bile acid signaling [42]. The relationship between the differential hepatic and cecum contents metabolites in bile acid pathway is summarized in Fig. 5. In humans, primary bile acids are CA and CDCA, which are converted by gut microbiota to secondary bile acids, such as LCA, DCA, and UDCA. Secondary bile acids have been reported to affect lipid metabolism, such as energy expenditure, insulin sensitivity, and cholesterol synthesis through farnesoid X receptor (FXR) and takeda-G-protein-receptor-5 (TGR5) pathways [43–45]. FXR signaling activation inhibits lipogenesis and promotes fatty acid oxidation and also affects cholesterol transportation [46, 47]. Bile acids differ in their potency to activate FXR and TGR5. In the present work, the results demonstrated that the concentrations of total bile acid were elevated in the liver and cecum, which is in accordance with the finding that obesity is associated with enhanced bile acid synthesis and impaired transport [48]. Increased bile acids production in patients are also associated with NAFLD, diabetes and metabolic syndrome [49–51]. Mouzaki et al. concluded that bile acid production in the livers of NAFLD was enhanced, based on the findings that faecal total bile acid and faecal primary bile acid levels in patients with NAFLD were increased [52]. This result was also supported by an epidemiological study which demonstrated that a low-fat, high-carbohydrate, and high-fiber diet consumption reduced fecal concentrations of secondary bile acid [53].

A few changes to primary and secondary metabolites in both liver and cecum contents between the lean and obese mice confirmed that lipids primarily drove this metabolic pathology. Cholic acid and chenodexycholic acid, known as primary bile acids, are synthesized from cholesterol in the liver and enter the small intestine where they are conjugated to glycine and taurine. Cholic acid (CA), the major bile acid, was significantly decreased 0.62-fold in the liver, which is inconsistent with the study that CA was increased 68-fold after high fat, high cholesterol, and cholate diet in mice [54]. The result was in harmony with the finding that liver level of cholic acid was significantly decreased in patients with NASH [55]. CA is a signaling molecule that activates the FXR-FGF15 pathway [56]. However, CA in cecum showed opposite results and increased 4.55-fold. High level of CA in cecum may be due to the absorption of cholate present in the high-fat diet, but it may also generated by the conversion of cholesterol to CA as a mechanism for excreting excessive cholesterol through gut-liver axis. Elevated chenodeoxycholic acid (CDCA) in the liver, which has been identified as effective ligands for FXR [41], was consistent with the report by Aranha et al. [57]. Taurochenodesoxycholic acid (TCDCA) was enhanced 24.36 and 13.71-fold in liver and cecum, respectively, is formed by conjugation of chenodeoxycholic acid (CDCA) with taurine in liver. The increased concentration of TCDCA in obese mice might be associated the disorder of liver function. The previous report indicated that TCDCA was enhanced in serum of liver cirrhosis and hepatocellular carcinoma patients [58]. Lithocholic acid (LCA) was reported to a hepatotoxic microbial metabolite [59]. Accordingly, taurolithocholic acid was elevated 4.36-fold in the liver, and lithocholytaurine was enhanced 13.57-fold in the cecum. The UDCA in the gut or the activation of the intestinal FXR pathway increases the expression of genes related to intestinal barrier function, thus benefiting the treatment of obesity and liver disease [60, 61]. Besides, UDCA was reported to improve hepatic steatosis and enhance insulin sensitivity by inducing the excretion of hepatic lipids, inhibiting hepatic long-chain FFA uptake, restoring gut mucosal integrity, and suppressing the miR-34a/SIRT1/p53 pathway in obese mice [61–63]. Our research consistently found that UDCA was significant reduced in obese mice. Thus, the change in bile acid metabolism induced by HFD plays multiple roles in regulating lipid metabolites in obese mice. However, more studies are required to clarify the role of specific bile acid in bile acid pool as a potential mechanism in obesity and metabolic disorders.

Furthermore, the level of oleic acid was also affected by HFD. Oleic acid is a monounsaturated fatty acid, which was enhanced 3.89-fold in the liver of obese mice. This result is consistent with the finding that oleic acid was significantly enhanced in serum of liver cirrhosis and hepatocellular carcinoma patients [58]. Alpha-linolenic acid (ALA) is an essential fatty acid, which was significantly lowered by HFD in liver. The result is in harmony with the research that alpha-linolenic acid-enriched butter attenuated high fat diet-induced insulin resistance and inflammation [63]. Accordingly, the levels of adrenic acid, icosenoic acid, and stearic acid involving in biosynthesis of unsaturated fatty acid in the cecum were significantly enhanced. The data is consistent with the report that higher proportion of stearic acid in serum phospholipids [64]. These results indicated that HFD intake had regulating effect on these metabolites involved in fatty acid metabolisms.

Most studies used male mice to construct high-fat diet-induced obesity model [12, 65, 66]. In the study, we used female mice in order to discover different outcomes in males based on their different hormonal profile. The results shared some similarities with previous studies that compared to the lean mice, a high-fat increased lipid profiles in the liver [12], and total bile acids (BAs) were enhanced in fecal metabolites in obese male mice [66]. However, the result was not in consistent with the finding that glycerol 3-phosphate and choline were significantly reduced [22], and CA was significantly elevated in the liver of male mice [54]. The specific mechanism that causes the different results in female and male mice needs further investigation. We proposed metabolic pathways associated with high-fat diet-induced obesity based on the metabolites we found in this study (Figs. 4, 5). However, the causal relationship between these identified biomarkers and the exact underlying mechanisms of the metabolites changes in obesity are still unclear. Furthermore, a large number of markers were detected by UHPLC-Q-TOF/MS but remain unidentified at present. Despite these limitations, the present study showed a cluster of obesity-associated alterations in metabolites, the proposed pathway will presumably facilitate our understanding of obesity and associated diseases symptoms.

## Conclusions

The UPLC-Q-TOF MS-based liver and cecum contents studies have been performed to reveal the complex interactions between liver and gut of obese mice induced by a high-fat diet and provided important metabolic information for obesity and related diseases. A high-fat diet increased lipid profiles (i.e. glycerophospholipids, PC, PE, PI, PG, and PS) and total bile acid (primary and secondary bile acid) in liver and cecum, indicating that HFD regulated glycerophospholipid metabolism, primary bile acid synthesis, and fatty metabolism pathways. This study provides evidence regarding the important regulatory functions of metabolites produced by gut commensal bacteria as well as insights into the interaction between the microbial products and host. The differences in these metabolic profiles between the obese and lean mice may provide a better understanding of the metabolic changes of obesity, which could be used for future clinical diagnosis and treatment.

## Data Availability

All data generated or analyzed during this study are included in this published paper or available from the corresponding author on reasonable request.

## Abbreviations

BCAAs: Branched-amino acids
CA: Cholic acid
CDCA: Chenodeoxycholic acid
CDP-choline: Cytidine 5′-diphosphocholine
ESI+: Electrospray ionization in positive ion mode
ESI−: Electrospray ionization in negative ion mode
HFD: High-fat diet
LC–MS: Liquid chromatography–mass spectrometry
LysoPC: Lysophophatidylcholine
lysoPE: Lysophosphatidylethanolamine
NAFLD: Nonalcoholic fatty liver disease
OPLS-DA: Orthogonal partial least squares discriminant analyses
PC: Phosphatidylcholine
PE: Phosphoethanolamine
PG: Phosphatidylglycerol
PI: Phosphatidyl inositol
PS: Phosphatidylserine
RT: Retention time
TCA: Taurocholic acid
TCDCA: Taurochenodeoxycholic acid
TDCA: Taurodeoxycholic acid
TG: Triglyceride
TLCA: Taurolithocholic acid
TUDCA: Tauroursodeoxycholic acid
UDCA: Ursodeoxycholic acid
UHPLC-Q-TOF/MS: Ultraperformance liquid chromatography-quadrupole-time-of-flight mass spectrometry
VIP: Variance importance for projection

## References

[1] Reilly JJ (2017). Health effects of overweight and obesity in 195 countries over 25 years. N Engl J Med.

[2] Ying Z, Hiroko K, Sei K (2018). Add-on therapy with traditional Chinese medicine: an efficacious approach for lipid metabolism disorders. Pharmacol Res.

[3] Wagstaff LR, Mitton MW, Arvik ML, Doraiswamy PM (2003). Statin-associated memory loss: analysis of 60 case reports and review of the literature. Pharmacotherapy.

[4] William R, Wikoff AT, Jun L, Peter GS, Scott AL, Eric CP (2009). Metabolomics analysis reveals large effects of gut microflora on mammalian blood metabolites. Proc Natl Acad Sci USA.

[5] Marshall JC (1998). The gut as a potential trigger of exercise-induced inflammatory responses. Can J Physiol Pharmacol.

[6] Sprockett D, Fukami T, Relman DA (2018). Role of priority effects in the early-life assembly of the gut microbiota. Nat Rev Gastroenterol Hepatol.

[7] Compare D, Coccoli P, Rocco A, Nardone OM, Maria SD, Cartenì M (2012). Gut-liver axis: the impact of gut microbiota on non-alcoholic fatty liver disease. Nutr Metab Cardiovasc Dis.

[8] Gakuhei S, Michael K, Hines IN (2010). Contribution of gut bacteria to liver pathobiology. Gastroenterol Res Pract.

[9] Rooks MG, Garrett WS (2016). Gut microbiota, metabolites and host immunity. Nat Rev Immunol.

[10] Lee WJ, Hase K (2014). Gut microbiota-generated metabolites in animal health and disease. Nat Chem Biol.

[11] Husted AS, Trauelsen M, Rudenko O, Hjorth SA, Schwartz TW (2017). GPCR-mediated signaling of metabolites. Cell Metab.

[12] Kim HJ, Kim JH, Noh S, Hur HJ, Sung MJ, Hwang JT (2011). Metabolomic analysis of livers and serum from high-fat diet induced obese mice. J Proteome Res.

[13] Robertson DG, Frevert U (2013). Metabolomics in drug discovery and development. Clin Pharmacol Ther.

[14] Wilson ID, Plumb R, Granger J, Major H, Williams R, Lenz EM (2005). HPLC-MS-based methods for the study of metabolomics. J Chromatogr B Anal Technol Biomed Life.

[15] Cai H, Wen Z, Li X, Meng K, Yang P (2020). *Lactobacillus plantarum* FRT10 alleviated high-fat diet-induced obesity in mice through regulating the PPARα signal pathway and gut microbiota. Appl Microbiol Biotechnol.

[16] Yin X, Peng J, Zhao L, Yu Y, Zhang X, Liu P (2013). Structural changes of gut microbiota in a rat non-alcoholic fatty liver disease model treated with a Chinese herbal formula. Syst Appl Microbiol.

[17] Si GLR, Yao P, Shi L (2015). Rapid determination of bile acids in bile from various mammals by reversed-phase ultra-fast liquid chromatography. J Chromatogr Sci.

[18] Zeisel SH (1992). Choline: an important nutrient in brain development, liver function and carcinogenesis. J Am Coll Nutr.

[19] Zeisel SH, Blusztajn JK (1994). Choline and human nutrition. Annu Rev Nutr.

[20] Leung C, Rivera L, Furness JB, Angus PW (2016). The role of the gut microbiota in NAFLD. Nat Rev Gastroenterol Hepatol.

[21] Imajo K, Fujita K, Yoneda M, Shinohara Y, Suzuki K, Mawatari H (2012). Plasma free choline is a novel non-invasive biomarker for early-stage non-alcoholic steatohepatitis: a multi-center validation study. Hepatol Res.

[22] Park H, Hur HJ, Kim S, Park S, Hong MJ, Sung MJ (2016). Biochanin A improves hepatic steatosis and insulin resistance by regulating the hepatic lipid and glucose metabolic pathways in diet-induced obese mice. Mol Nutr Food Res.

[23] Mohsen M, Niki K, Dimitri PM, Maciej B (2019). Adiposity may moderate the link between choline intake and non-alcoholic fatty disease. J Am Coll Nutr.

[24] Rinella ME, Elias MS, Smolak RR, Fu T, Borensztajn J (2008). Mechanisms of hepatic steatosis in mice fed a lipogenic methionine choline-deficient diet. J Lipid Res.

[25] Rinella ME, Green RM (2004). The methionine-choline deficient dietary model of steatohepatitis does not exhibit insulin resistance. J Hepatol.

[26] Puri P, Baillie RA, Wiest MM, Mirshahi F, Choudhury J, Cheung O (2007). A lipidomic analysis of nonalcoholic fatty liver disease. Hepatology.

[27] Shirouchi B, Nagao K, Inoue N, Ohkubo T, Hibino H, Yanagita T (2007). Effect of dietary omega 3 phosphatidylcholine on obesity-related disorders in obese otsuka long-evans tokushima fatty rats. J Agric Food Chem.

[28] Noga AA, Vance DE (2003). Insights into the requirement of phosphatidylcholine synthesis for liver function in mice. J Lipid Res.

[29] Kawano Y, Nishiumi S, Saito M, Yano Y, Azuma T, Yoshida M (2015). Identification of lipid species linked to the progression of non-alcoholic fatty liver disease. Curr Drug Targets.

[30] Meikle PJ, Summers SA (2016). Sphingolipids and phospholipids in insulin resistance and related metabolic disorders. Nat Rev Endocrinol.

[31] Siddiqi S, Mansbach CM (2015). Dietary and biliary phosphatidylcholine activates PKCζ in rat intestine. J Lipid Res.

[32] Annika B, Irina T, Daniel G, Anke T, Alexandra Z, Rebecca R (2009). Alterations of phospholipid concentration and species composition of the intestinal mucus barrier in ulcerative colitis: a clue to pathogenesis. Inflamm Bowel Dis.

[33] Benno Y, Endo K, Mizutani T, Namba Y, Mitsuoka T (1989). Comparison of fecal microflora of elderly persons in rural and urban areas of Japan. Appl Environ Microbiol.

[34] Ma N, Liu X, Kong X, Li S, Jiao Z, Qin Z (2018). Aspirin eugenol ester regulates cecum contents metabolomic profile and microbiota in an animal model of hyperlipidemia. BMC Vet Res.

[35] Deng Y, Pan M, Nie H, Zheng C, Tang K, Zhang Y (2019). Lipidomic analysis of the protective effects of Shenling Baizhu San on non-alcoholic fatty liver disease in rats. Molecules.

[36] Oak JH, Nakagawa K, Miyazawa T (2002). UV analysis of amadori-glycated phosphatidylethanolamine in foods and biological samples. J Lipid Res.

[37] Oak JH, Nakagawa K, Miyazawa T (2000). Synthetically prepared amadori-glycated phosphatidylethanolamine can trigger lipid peroxidation via free radical reactions. FeEBS Lett.

[38] Nie J, Hao X, Chen D, Han X, Chang Z, Shi Y (2010). A novel function of the human CLS1 in phosphatidylglycerol synthesis and remodeling. Biochem Biophys Acta.

[39] KawasakiY K, Kuge O, Chang SC, Heacock PN, Rho M, Suzuki K (1999). Isolation of a Chinese hamster ovary (CHO) cDNA encoding phosphatidylglycerophosphate (PGP) synthase, expression of which corrects the mitochondrial abnormalities of a PGP synthase-defective mutant of CHO-K1 cells. J Biol Chem.

[40] Chiang JYL, Ferrell JM (2019). Bile acids as metabolic regulators and nutrient sensors. Annu Rev Nutr.

[41] de Aguiar Vallim TQ, Tarling EJ, Edwards PA (2013). Pleiotropic roles of bile acids in metabolism. Cell Metab.

[42] Ridlon JM, Kang D, Hylemon PB (2006). Bile salt biotransformations by human intestinal bacteria. J Lipid Res.

[43] Boer JF, Bloks VW, Verkade E, Heiner-Fokkema MR, Kuipers F (2018). New insights in the multiple roles of bile acids and their signaling pathways in metabolic control. Curr Opin Lipidol.

[44] Jia W, Xie G, Jia W (2018). Bile acid–microbiota crosstalk in gastrointestinal inflammation and carcinogenesis. Nat Rev Gastroenterol Hepatol.

[45] Moran-Ramos S, López-Contreras BE, Canizales-Quinteros S (2017). Gut microbiota in obesity and metabolic abnormalities: a matter of composition or functionality?. Arch Med Res.

[46] Watanabe M, Houten SM, Wang L, Moschetta A, Mangelsdorf DJ, Heyman RA (2004). Bile acids lower triglyceride levels via a pathway involving FXR, SHP, and SREBP-1c. J Clin Investig.

[47] Chao F, Gong W, Zheng Y, Li Y, Huang G, Gao M (2010). Upregulation of scavenger receptor class B type I expression by activation of FXR in hepatocyte. Atherosclerosis.

[48] Haeusle RA, Stefania C, Monica N, Brenno A, Jose CP, Dan X (2016). Increased bile acid synthesis and impaired bile acid transport in human obesity. J Clin Endocrinol Metab.

[49] Cariou B, Chetiveaux M, Zaïr Y, Pouteau E, Disse E, Guyomarc'h-Delasalle B (2011). Fasting plasma chenodeoxycholate and cholic acid concentrations are inversely correlated with insulin sensitivity in adults. Nutr Metab (Lond).

[50] Haeusler RA, Astiarraga B, Camastra S, Accili D, Ferrannini E (2013). Human insulin resistance is associated with increased plasma levels of 12-hydroxylated bile acids. Diabetes.

[51] Brufau G, Stellaard F, Prado K, Bloks VW, Jonkers E, Boverhof R (2010). Improved glycemic control with colesevelam treatment in patients with type 2 diabetes is not directly associated with changes in bile acid metabolism. Hepatology.

[52] Marialena M, Wang AY, Robert B, Comelli EM, Arendt BM, Zhang L (2016). Bile acids and dysbiosis in non-alcoholic fatty liver disease. PLoS ONE.

[53] Reddy BS, Engle A, Simi B, O”Brien LT, Barnard RJ, Pritikin N (1988). Effect of low-fat, high-carbohydrate, high-fiber diet on fecal bile acids and neutral sterols. Prev Med.

[54] Tu LN, Showalter MR, Cajka T, Fan S, Pillai VV, Fiehn O (2017). Metabolomic characteristics of cholesterol-induced non-obese nonalcoholic fatty liver disease in mice. Sci Rep.

[55] Han JH, Dzierlenga AL, Lu Z, Billheimer DD, Torabzadeh E (2017). Metabolomic profiling distinction of human nonalcoholic fatty liver disease progression from a common rat model. Obesity.

[56] Inagaki T, Choi M, Moschetta A, Li P, Cummins CL, McDonald JG (2005). Fibroblast growth factor 15 functions as an enterohepatic signal to regulate bile acid homeostasis. Cell Metab.

[57] Aranha MM, Cortez-Pinto H, Costa A, Silva IB, Camilo ME, Moura MC (2008). Bile acid levels are increased in the liver of patients with steatohepatitis. Eur J Gastroenterol Hepatol.

[58] Yin P, Wan D, Zhao C, Chen J, Zhao X, Wang W (2009). A metabonomic study of hepatitis B-induced liver cirrhosis and hepatocellular carcinoma by using RP-LC and HILIC coupled with mass spectrometry. Mol Biosyst.

[59] Pathak P, Xie C, Nichols RG, Ferrell JM, Boehme S, Krausz KW (2018). Intestine farnesoid X receptor agonist and the gut microbiota activate G-protein bile acid receptor-1 signaling to improve metabolism. Hepatology.

[60] Gadaleta RM, Erpecum KJV, Oldenburg B, Willemsen ECL, Renooij W, Murzilli S (2011). Farnesoid X receptor activation inhibits inflammation and preserves the intestinal barrier in inflammatory bowel disease. Gut.

[61] Golden JM, Escobar OH, Nguyen MVL, Mallicote MU, Kavarian P, Frey MR (2018). Ursodeoxycholic acid protects against intestinal barrier breakdown by promoting enterocyte migration via EGFR- and COX-2-dependent mechanisms. Am J Physiol Gastrointest Liver Physiol.

[62] Quintero P, Pizarro M, Solís N, Arab JP, Padilla O, Riquelme A (2014). Bile acid supplementation improves established liver steatosis in obese mice independently of glucagon-like peptide-1 secretion. J Physiol Biochem.

[63] Nie B, Park HM, Kazantzis M, Lin M, Henkin A, Ng S (2012). Specific bile acids inhibit hepatic fatty acid uptake in mice. Hepatology.

[64] Kim JY, Park JY, Kim OY, Ham BM, Kim H, Kwon DY (2010). Metabolic profiling of plasma in overweight/obese and lean men using ultra performance liquid chromatography and Q-TOF mass spectrometry (UPLC-Q-TOF MS). J Proteome Res.

[65] Li Y, Cui Y, Hu X, Liao X, Zhang Y (2019). Chlorophyll supplementation in early life prevents diet-induced obesity and modulates gut microbiota in mice. Mol Nutr Food Res.

[66] Xu J, Li M, Zhang Y, Chu S, Huo Y, Zhao J (2020). Huangjinya black tea alleviates obesity and insulin resistance via modulating fecal metabolome in high-fat diet-fed mice. Mol Nutr Food Res.

